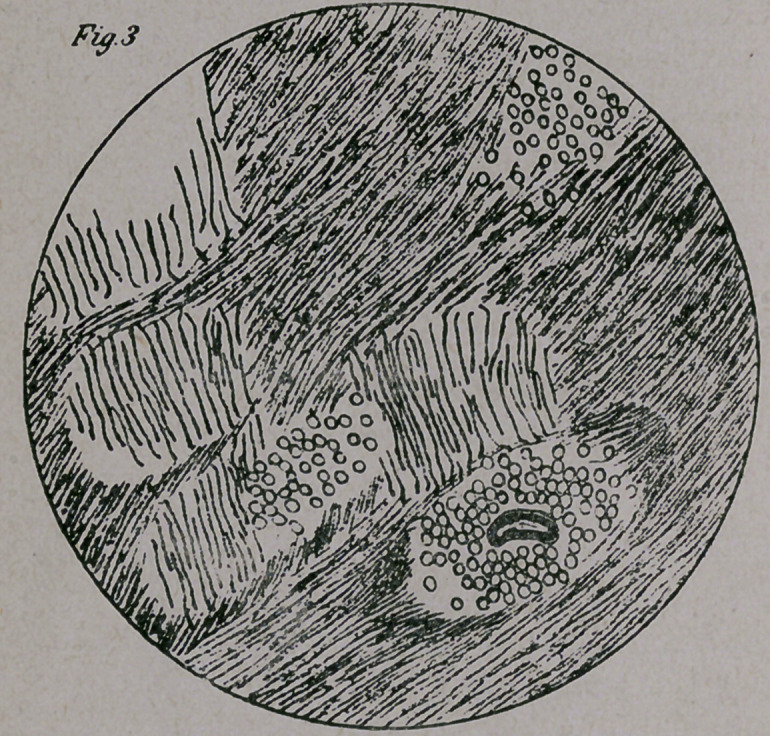# On the Pathological Anatomy of Diphtheritic Paralyses

**Published:** 1889-01

**Authors:** William C. Krauss

**Affiliations:** Attica, N. Y.; Laboratory of Prof. Mendel, Berlin; 5 Rue Rollin, Paris


### *ON THE PATHOLOGICAL ANATOMY OF DIPHTHERITIC PARALYSES.*

By Dr. WILLIAM C. KRAUSS, Attica, N. Y.

Laboratory of Prof. Mendel, Berlin.

Translated for *Buffalo Medical and Surgical Journal*, by the Author.

[From *Neurologisches Centralblatt, Leipzig*, 1888, No. 16.]

The diversity of opinion held by various authors in regard to the pathological anatomy of diphtheritic paralyses, and the small number of authenticated cases which have been published on this subject, has led me to examine carefully the following case, hoping it may help to solve the problem of these paralyses:

Gertrude K., eleven years old, was taken sick October 15, 1887, with symptoms of a severe diphtheritis, which continued until November 1, 1887, when exitus letalis occurred.

The autopsy made the following day revealed the following:

Heart: Several large firm thrombi.

Lungs: Edematous.

Liver, kidneys, spleen offer nothing abnormal.

In the ventral cavity about 500 c. cm. of a sero-fibrinous fluid.

Intestines: Somewhat injected and adherent.

Diagnosis: Diphtheritis, et peritonitis recens sero-fibrinosa.

[NOTE.—The history of the case and results of the autopsy are taken from the reports at the Moabit Krankenhaus, Berlin.]

The macroscopic examination of the brain shows a marked hyperemia; the pia is easily removed; no adhesions; no thickening of the same; a section through the cerebrum shows èdema and engorgement of the blood-vessels.

The base of the cerebrum from the crura cerebri to the decussation of the pyramids, containing the nuclei of the cranial nerves from the third to the twelfth inclusive, was hardened in a three per cent. solution of bichromate of potassium, decolorized and dehydrated in alcohol, and imbedded in pelloidin. A series of 650 consecutive sections was made and prepared. The staining methods employed were: Hematoxylin, ammonia-carmine, picro-carmine, nigrosine—the Pal, and the Weigert methods.

The special object of the examination was chiefly to determine whether any changes had taken place: First, in the nuclei of the oculomotorius, abducens facial and hypoglossal nerves; second, the intracerebral roots of the same; and third, the blood-vessels.

The examination showed the following changes:

The ganglionic cells of the various nuclei were found in a normal condition, no appreciable changes having occurred either in their number, size, form, or contents.

The nerve fibers were found to be the seat of several important changes, and especially those of the intra-cerebral oculo-motorius. A small number of axis cylinders were found destroyed; some showed irregularity in size, while others had lost their sharp contours. The myelinic sheaths had in part taken the staining fluid, an evidence of the loss of their normal chemical composition.

This change was noted particularly in those preparations which had been stained with ammonia-carmine and picro-carmine.

Fig. 1.

Fig. 1. A cross-section of the intra-cerebral oculo-motorius shows plainly the changes just mentioned.

The blood-vessels presented important and striking changes. The capillaries, smaller as well as larger arteries, were found in a state of engorgement. So marked was this vascularization, that the preparations gave the appearance of a vascular new-growth. On the other hand, the veins in the floor of the fourth ventricle were found in an empty condition, their walls collapsed. The hyperemia was pretty regular, and could be noticed in every preparation of the whole series.

The next important change was a remarkable diapedesis of the blood-corpuscles, due, no doubt, to finer changes in the walls of the blood-vessels. This change is now generally accepted as due to the diphtheritic virus causing slight inflammation of the vessel walls.

*Fig. 2.*

Fig. 2. A cross-section through the pons near the trigeminus shows the condition of a blood-vessel. One sees along the entire length of the blood-vessel the diapedesis of the blood corpuscles, and some, further in the tissues of the pons.

This diapedesis was noticeable in nearly every section, and was most striking in those sections stained with nigrosine. In these sections, the blood corpuscles, stained yellow by the hardening fluid — bichromate of potassium—refused to accept the nigrosine, thus showing a marked contrast to the vessel-walls, stained blue by the nigrosine.

The examination showed, further, a large number of small hemorrhages which had taken place in the pons. The results of the diapedeses were accumulations of blood corpuscles in the perivascular spaces.

Fig. 2 shows the diapedesis and the accumulation of blood corpuscles in the perivascular spaces.

The greater hemorrhages were, in part, visible to the naked eye, especially near the intra-pontile course of the nerves, and near the origin of the left oculo-motorius. Hemorrhages had also taken place along the roots of the oculo-motorius, and also at the sulcus oculo-motorius; also along the intra-pontile course of the abducens and the trigeminus. The roots of the other nerves presented nothing abnormal.

A large hemorrhage had also taken place at the trigonum inter-pedancular, surrounding the roots of the oculo-motorius. This hemorrhage, which had taken considerable proportions, was not, however, sufficient to call forth symptoms of apoplexia sanguinea—resp. death.

The examination showed no traces of thrombi or emboli.

Fig. 3

Fig. 3 shows part of the intra-pontile fibers of the abducens nerve, some having been pushed aside, and some destroyed, by the hemorrhages along its course.

The summary of this examination shows:

1. Normal nuclei of cranial nerves.

2. Degeneration in part of the oculo-motorious nerve.

3. Hyperemia, diapedesis of the blood corpuscles, and hemorrhages of various proportions.

The results obtained agree, in general, with those of Mendel, published in the *Neurologische Centralblatt*, 1885, No. 6.

To the pathological anatomy of diphtheritic paralyses nothing has been added since the publication of Mendel's case. A compilation of previous observations on this subject is to be found in *Virchow's Archiv für Pathologische Anatomiè*, vol. lxxxv., p. 214, by Paul Meyer.

In conclusion, I tender to Prof. Mendel my hearty thanks for the kindnesses shown me in the preparation of this paper, as well as for materials used.

5 RUE ROLLIN, PARIS.

## Figures and Tables

**Fig. 1. f1:**
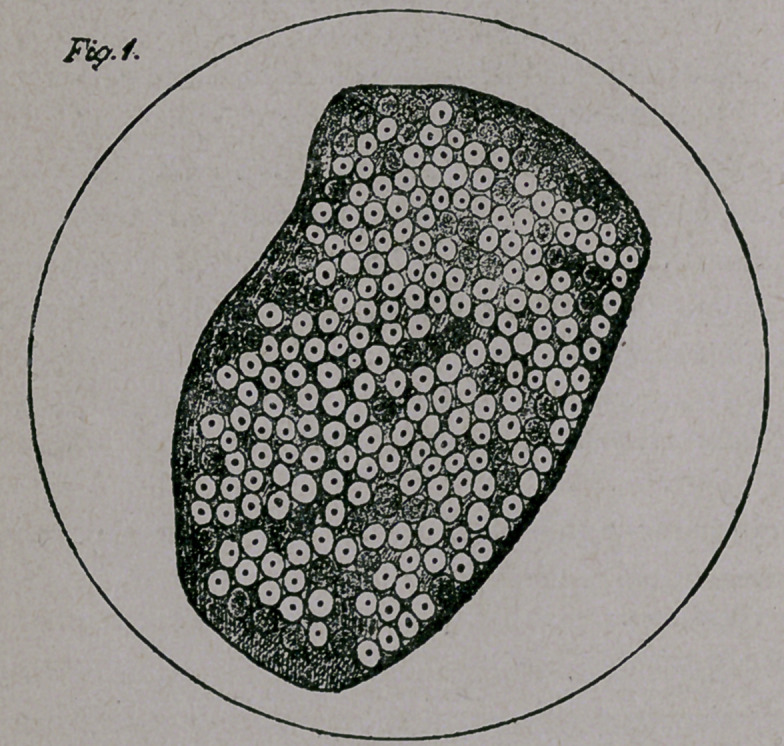


**Fig. 2. f2:**
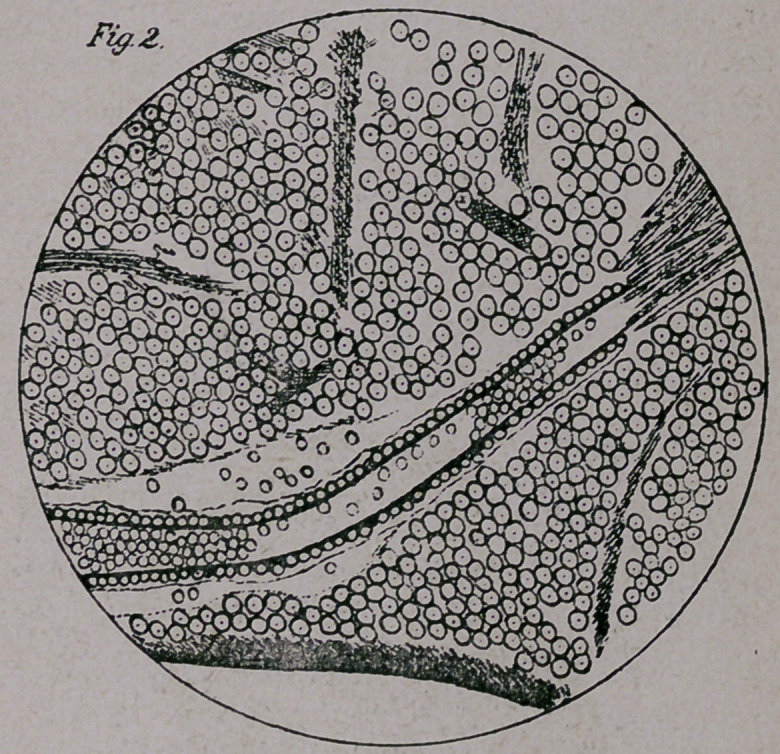


**Fig. 3. f3:**